# CK1ε Is Required for Breast Cancers Dependent on β-Catenin Activity

**DOI:** 10.1371/journal.pone.0008979

**Published:** 2010-02-01

**Authors:** So Young Kim, Ian F. Dunn, Ron Firestein, Piyush Gupta, Leslie Wardwell, Kara Repich, Anna C. Schinzel, Ben Wittner, Serena J. Silver, David E. Root, Jesse S. Boehm, Sridhar Ramaswamy, Eric S. Lander, William C. Hahn

**Affiliations:** 1 Department of Medical Oncology, Dana-Farber Cancer Institute, Boston, Massachusetts, United States of America; 2 Department of Neurosurgery, Brigham and Women's Hospital and Harvard Medical School, Boston, Massachusetts, United States of America; 3 Department of Pathology, Brigham and Women's Hospital and Harvard Medical School, Boston, Massachusetts, United States of America; 4 Department of Medicine, Brigham and Women's Hospital and Harvard Medical School, Boston, Massachusetts, United States of America; 5 Center for Cancer Genome Discovery, Dana-Farber Cancer Institute, Boston, Massachusetts, United States of America; 6 Broad Institute, Cambridge, Massachusetts, United States of America; 7 Massachusetts General Hospital Cancer Center, Boston, Massachusetts, United States of America; 8 Department of Medicine, Harvard Medical School, Boston, Massachusetts, United States of America; 9 Harvard Stem Cell Institute, Cambridge, Massachusetts, United States of America; Roswell Park Cancer Institute, United States of America

## Abstract

**Background:**

Aberrant β-catenin signaling plays a key role in several cancer types, notably colon, liver and breast cancer. However approaches to modulate β-catenin activity for therapeutic purposes have proven elusive to date.

**Methodology:**

To uncover genetic dependencies in breast cancer cells that harbor active β-catenin signaling, we performed RNAi-based loss-of-function screens in breast cancer cell lines in which we had characterized β-catenin activity. Here we identify *CSNK1E*, the gene encoding casein kinase 1 epsilon (CK1ε) as required specifically for the proliferation of breast cancer cells with activated β-catenin and confirm its role as a positive regulator of β-catenin-driven transcription. Furthermore, we demonstrate that breast cancer cells that harbor activated β-catenin activity exhibit enhanced sensitivity to pharmacological blockade of Wnt/β-catenin signaling. We also find that expression of CK1ε is able to promote oncogenic transformation of human cells in a β-catenin-dependent manner.

**Conclusions/Significance:**

These studies identify CK1ε as a critical contributor to activated β-catenin signaling in cancer and suggest it may provide a potential therapeutic target for cancers that harbor active β-catenin. More generally, these observations delineate an approach that can be used to identify druggable synthetic lethal interactions with signaling pathways that are frequently activated in cancer but are difficult to target with the currently available small molecule inhibitors.

## Introduction

The Wnt/β-catenin pathway plays a critical role in embryonic development, maintenance of multipotent progenitor cell populations and proliferation of many tissue types [1], [2]. In the absence of Wnt ligands, a complex containing APC, AXIN and GSK3 phosphorylates β-catenin, marking it as a substrate for ubiquitination by β-TrCP and subsequent proteasomal degradation. Canonical Wnt/β-catenin signaling is initiated by binding of Wnt ligands to Frizzled (Fzd)-LRP5/6 receptor complexes, leading to inactivation of the destruction complex and stabilization of β-catenin. Once stabilized, β-catenin accumulates and translocates to the nucleus, where it complexes with TCF/LEF to activate transcription of target genes, such as *MYC* and *CCND1*. In addition to ligand-regulated degradation of β-catenin, Wnt signaling is antagonized by extracellular factors that inhibit the ability of Wnt ligands to bind to Fzd and initiate signaling, such as the secreted frizzled-related proteins (SFRP1, WNT inhibitory factor (WIF) and dickkopf (DKK) [3].

Loss-of-function mutations in *APC* or *AXIN* or activating mutations in the gene encoding β-catenin, *CTNNB1*, lead to aberrant activation of Wnt/β-catenin signaling and have been causally linked to tumorigenesis of the colon, liver and skin [1], [4]. Although mutations in these same genes have not been observed as recurrent genetic events in breast tumors, there is strong evidence implicating Wnt/β-catenin activity in breast tumorigenesis. Wnt1 was originally discovered as an oncogene activated by mouse mammary tumor virus (MMTV) [5], and mice engineered to express either Wnt1 or an activated form of β-catenin from the MMTV-LTR develop mammary hyperplasia and adenocarcinoma [6]. Moreover, human breast tumors frequently exhibit elevated levels of nuclear β-catenin, with higher expression levels correlating with decreased patient survival [7]. The mechanism of β-catenin activation in breast tumors appears to involve the downregulation of Wnt inhibitors, such as SFRP, WIF or DKK, leading to constitutive activation of autocrine Wnt signaling [8]. In the case of the SFRP genes and *WIF1*, this downregulation often occurs through methylation-induced epigenetic silencing [9], [10], and both *SFRP1* and *DKK* have been shown to be transcriptionally repressed by *MYC*, a well-established breast oncogene [11]. However, it remains unclear whether inactivation of these inhibitors fully explains the observed frequency of β-catenin activation in breast cancers.

Given the potentially important role of Wnt/β-catenin signaling in breast tumorigenesis, we sought to identify potential therapeutic targets, by identifying genes that promote the survival of breast cancer cells with active β-catenin. By using an RNAi-mediated loss-of-function screening approach, we have identified *CSNK1E*, a member of the casein kinase family, as an essential regulator of β-catenin activity and proliferation in breast cancer cell lines.

## Results

### Characterization of β-Catenin Activity and Dependency in Breast Cancer Cell Lines

**Figure 1:**
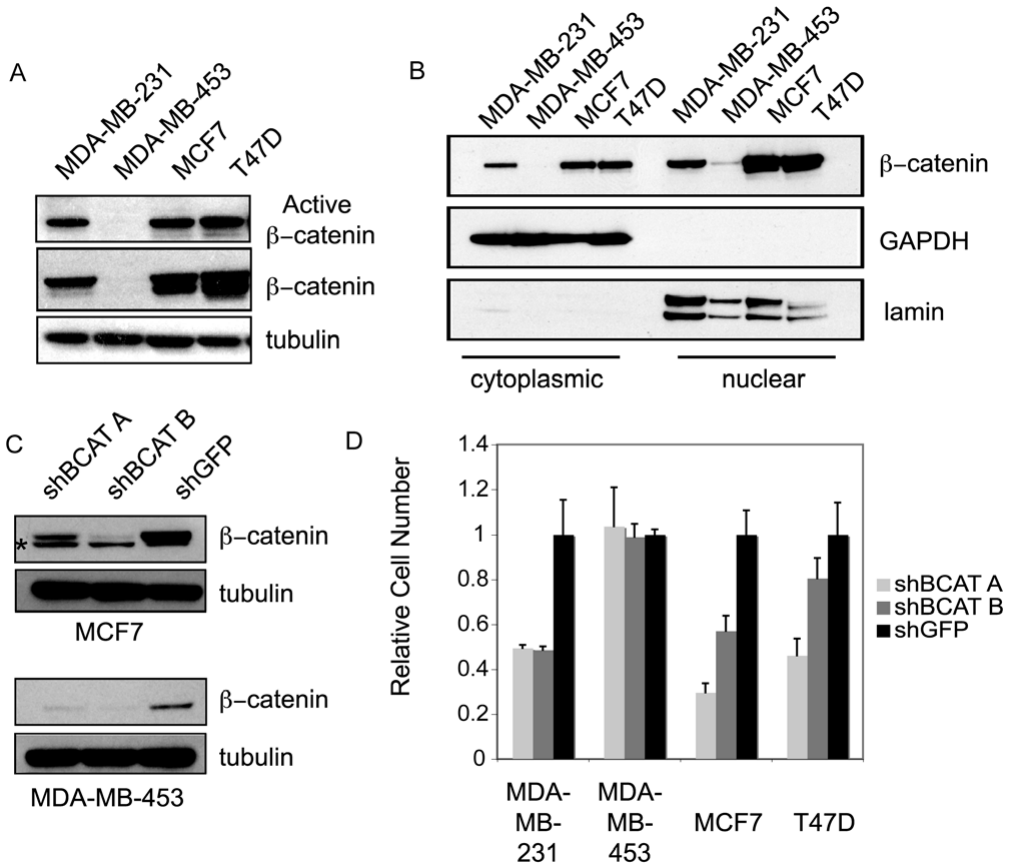
Characterization of Wnt/β-catenin activity in breast cancer cell lines. Immunoblot analysis of (A) active (upper panel) and total (middle panel) β-catenin levels or (B) cytoplasmic (left) and nuclear (right) β-catenin levels. To verify fractionation, immunoblots for cytoplasmic GAPDH and nuclear lamin are shown. (C) Immunoblot analysis of β-catenin levels after suppression of *CTNNB1* with two distinct shRNAs (shBCAT A, B) in β-catenin active (MCF7) and β-catenin inactive (MDA-MB-453) cells. An shRNA against GFP was included as a control (shGFP). Asterisk denotes position of a non-specific cross-reacting band. (D) Effects on proliferation after RNAi-induced suppression of *CTNNB1*. Graph shows mean ± SD of a representative experiment performed in triplicate.

We initially characterized the activation status of β-catenin in breast cancer cell lines using an antibody that specifically recognizes the unphosphorylated form of β-catenin, which corresponds to the stable and thus functionally active form [8]. Three of the lines tested, MCF7, MDA-MB-231 and T47D were found to have elevated levels of both active and total β-catenin compared with the MDA-MB-453 cell line, which had much lower levels (Fig. 1A). In addition to determining levels of the active form of β-catenin, we also assessed β-catenin activity status by measuring nuclear β-catenin levels, which corresponds to the transcriptionally active pool of β-catenin, and is physically and functionally separate from the other major cellular pool of β-catenin at adherens junctions. We observed elevated levels of nuclear β-catenin in MCF7, MDA-MB-231 and T47D cells, with barely detectable levels in the MDA-MB-453 cells. (Fig. 1B). Methylation and subsequent downregulation of expression of the Wnt-inhibitory gene *SFRP1* has previously been observed in all three β-catenin-expressing lines [12] and may contribute to activation of Wnt/β-catenin signaling in these cells.

We next determined the functional importance of β-catenin in these cells. When we suppressed *CTNNB1*, with two distinct short hairpin RNAs (shRNAs) in the three cell lines with active β-catenin, we observed a substantial reduction in cell proliferation (Fig. 1C, D). In contrast, suppression of β-catenin in MDA-MB-453 cells failed to alter proliferation (Fig. 1C, D). Taken together, these observations suggest that some breast cancer cells exhibit aberrant activation of β-catenin and further, that these cells are dependent on continued β-catenin function for proliferation.

### Loss-of-Function Screens Identify *CSNK1E* as an Essential Regulator of β-Catenin

**Figure 2:**
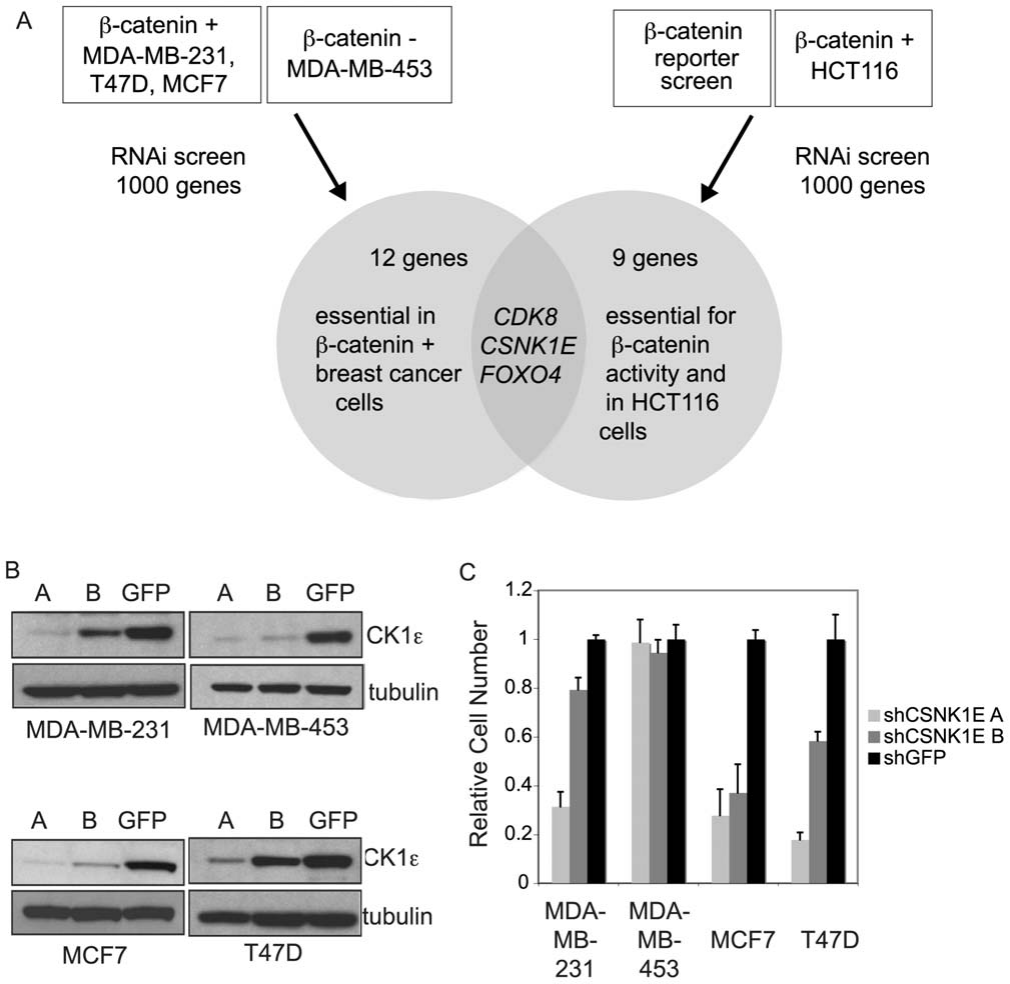
*CSNK1E* is an essential gene in breast cancer cells with active β-catenin. (A) Schematic overview of RNAi screens and integrative analysis to identify essential regulators of β-catenin activity and cancer cell proliferation. (B) Immunoblot analysis of CK1ε levels after RNAi-induced suppression. (C) Effects of *CSNK1E* suppression with two shRNA sequences (A and B) on proliferation. Graph shows mean ± SD of a representative experiment performed in triplicate.

After characterizing β-catenin activity, we performed high-throughput screening of the MCF7, MDA-MB-231, T47D and MDA-MB-453 breast cancer cell lines using a kinase-rich subset of the lentiviral shRNA library generated by the RNAi Consortium (http://www.broad.mit.edu/genome_bio/trc/rnai.html) to identify genes specifically required for proliferation of cells that harbor active β-catenin. We chose to focus on kinases as they regulate many key physiological processes and have the potential to rapidly translate to therapeutic targets thanks to the existence of readily available inhibitors. Raw luminescence scores derived from the proliferation/viability assay were normalized to plate medians and corrected for variability due to spatial and batch effects to generate B scores [13]. Replicates were averaged to generate a cumulative B score for each shRNA (Table S1). As the shRNA library provides redundant coverage of targeted genes, with approximately five shRNAs against each gene, we defined essential genes as those for which multiple shRNAs induced a reduction in proliferation, with at least two shRNAs with a B score below -1. Using this approach, we identified twelve genes, *STK6* (*AURKA*), *AURKB*, *CDK8*, *CSNK1E*, *DCK*, *KDR*, *FOXO4* (*MLLT7*), *PRKACA*, *PRKCA*, *STK16*, *TK1* and *VRK1* that were required for proliferation in the three cell lines that showed active β-catenin but not in the cell line with no evidence of β-catenin activation (Fig. 2A).

Based on our observations that β-catenin itself is required for proliferation in cells with active β-catenin, we hypothesized that some of these genes may affect proliferation through regulation of β-catenin activity. To pursue this possibility, we integrated the results of our proliferation screen with the results of a parallel screen performed using the same shRNA library to identify modulators of β-catenin transcriptional activity [14]. By comparing the results of these two screens, we found three genes to be essential for both proliferation and β-catenin activity, *FOXO4*, *CDK8* and *CSNK1E* (Fig. 2A). We recently characterized *CDK8* as a colorectal oncogene that functions as part of the Mediator complex to modulate β-catenin-driven transcription. Here, we focused on *CSNK1E*, a member of the casein kinase family that has been implicated in the regulation of circadian periodicity [15], as well as Wnt/β-catenin signaling [16], [17]. Interestingly, suppression of *CSNK1D*, which is highly homologous to and has overlapping function with *CSNK1E* [18], did not affect proliferation of β-catenin positive cancer cell lines, suggesting a specific role for *CSNK1E*, at least in the context of these breast cancer cells (Table S1).

We validated the ability of the shRNAs to reduce CK1ε levels and confirmed that two shRNA sequences that scored in the primary screens hits reduced the expression of CK1ε in each of the four cell lines screened (Fig. 2B). Importantly, these shRNAs only affected proliferation in the three cell lines that showed high β-catenin activity, recapitulating the phenotypes from the primary screen (Fig. 2C) and the ability of the individual shRNAs to affect proliferation correlated directly with their ability to reduce CK1ε levels (Fig. 2B, C).

**Figure 3:**
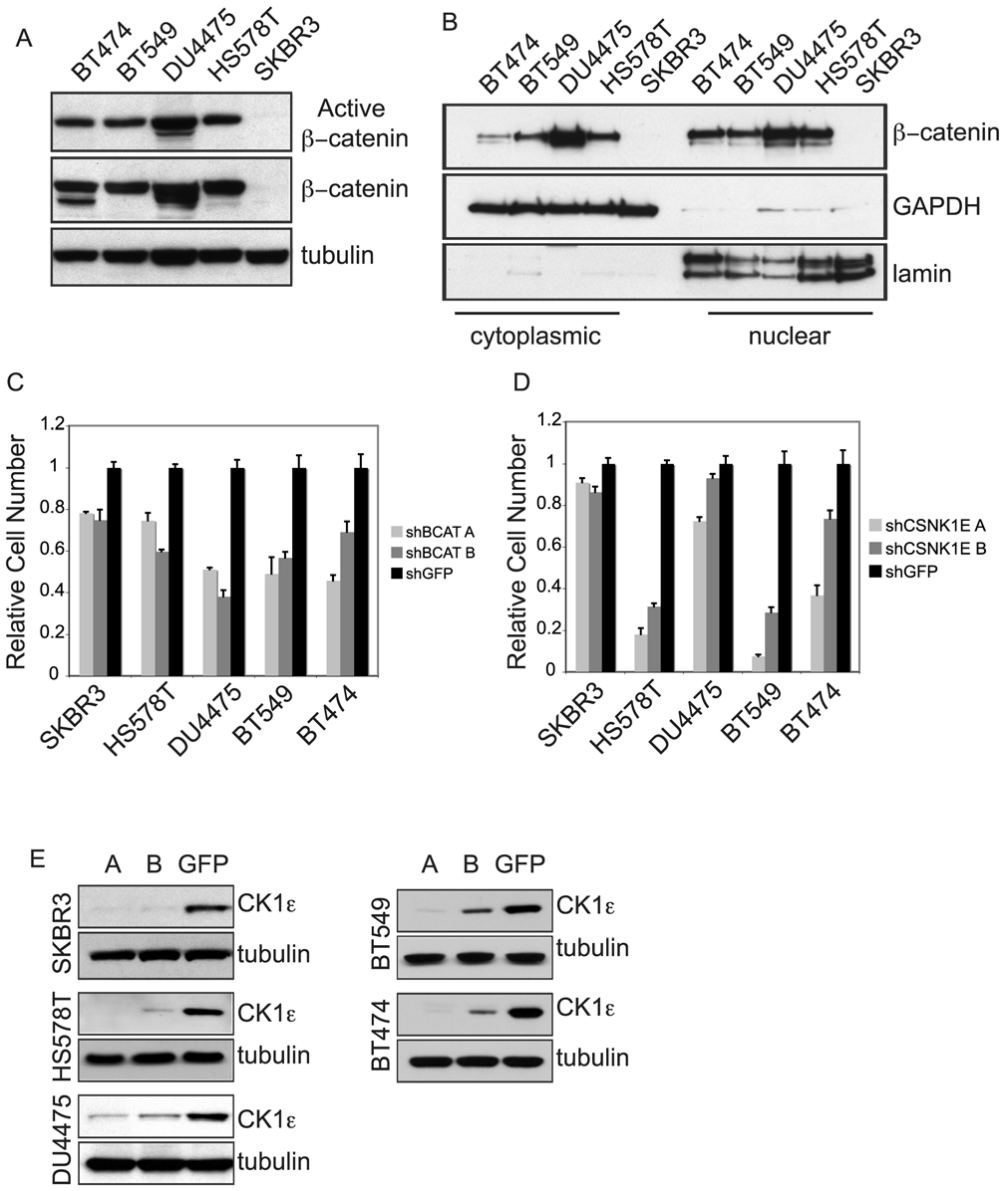
Validation of *CSNK1E* as an essential gene in additional β-catenin-activated breast cancer cells. (A) Immunoblot analysis of active (upper panel) and total (middle panel) β-catenin levels in five additional breast cancer cell lines, BT474, BT549, DU4475, HS578T, and SKBR3. (B) Immunoblot analysis of cytoplasmic (left) or nuclear (right) β-catenin levels. Immunoblots for cytoplasmic GAPDH and nuclear lamin were performed to verify fractionation. Effects of (C) *CTNNB1* or (D) *CSNK1E* suppression on proliferation. Graph shows mean ± SD of a representative experiment performed in triplicate. (E) Immunoblot analysis of CK1ε levels after RNAi-induced suppression.

As the initial RNAi screens were limited to three β-catenin-positive lines and one β-catenin-negative line, we tested the hypothesis that *CSNK1E* is preferentially required in additional β-catenin-positive cells by determining the effects of its suppression in an expanded panel of breast cancer cell lines. We assessed these cell lines for levels of unphosphorylated, active β-catenin (Fig. 3A), levels of nuclear β-catenin (Fig. 3B), and dependency on β-catenin (Fig. 3C) and identified four additional breast cancer cells with evidence of β-catenin activity (BT474, BT549, DU4475 and HS578T) and one additional β-catenin-negative line (SKBR3). These cell lines exhibited varying degrees of sensitivity to suppression of *CTNNB1*, with the β-catenin-negative line, SKBR3, being the least sensitive (Fig. 3C). Of particular interest, the DU4475 cell line has been reported to harbor a homozygous nonsense mutation in the *APC* gene [19], leading to the increased levels of active and total β-catenin observed (Fig. 3A, B). These cell lines were then tested for their response to the loss of *CSNK1E* function. We observed that suppression of *CSNK1E* led to reduced proliferation in three of the β-catenin-positive lines, HS578T, BT474, and BT549 but did not significantly affect the β-catenin-negative line, SKBR3 (Fig. 3D, E). Interestingly, the *APC*-mutated DU4475 cells, while exhibiting evidence of β-catenin activation and sensitivity to *CTNNB1* suppression, were relatively unaffected by *CSNK1E* suppression (Fig. 3D, E), suggesting that *CSNK1E* may function upstream of *APC* in Wnt/β-catenin signaling.

### Small Molecule Inhibition of CK1ε

**Figure 4:**
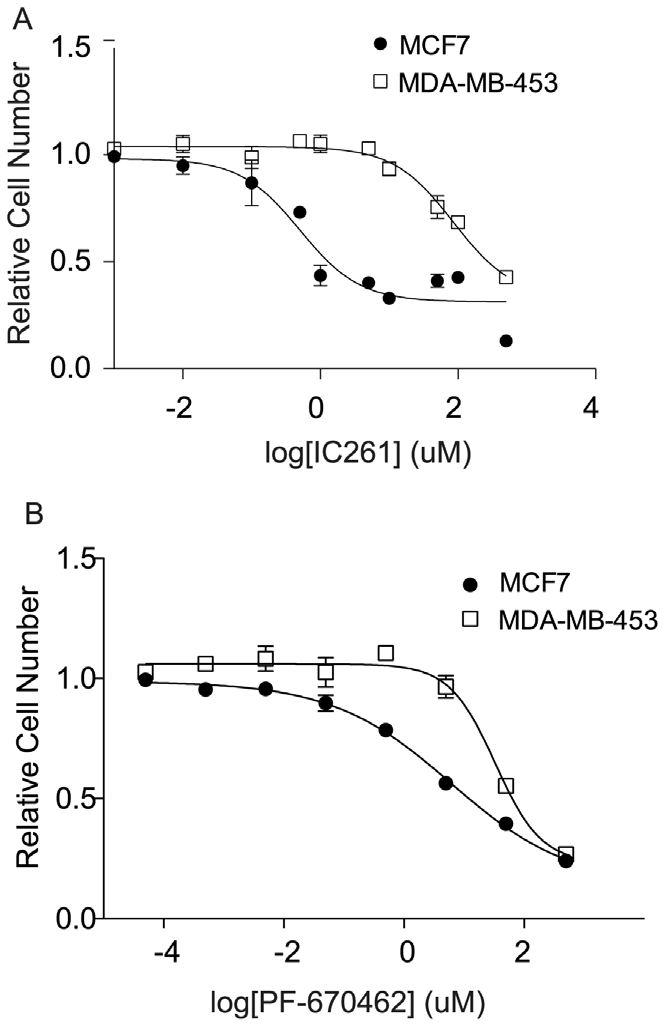
Small molecule inhibition of CK1ε specifically affects breast cancer cells with β-catenin activity. (A) IC261 dose response curves of β-catenin active cells (MCF7) and β-catenin inactive cells (MDA-MB-453). (B) PF-670462 dose response curves of β-catenin active cells (MCF7) and β-catenin inactive cells (MDA-MB-453). Graphs show mean ± SD of a representative experiments performed in triplicate.

To evaluate the role of *CSNK1E* as an essential gene in β-catenin-positive cell lines using a second independent approach, we used IC261, a specific inhibitor of CK1ε. Upon treatment with IC261, we observed a reduction in proliferation in β-catenin-positive MCF7 cells, with an IC50 of 0.5 uM, which closely parallels the reported IC50 for the inhibition of CK1ε by IC261 (1 uM) (Fig. 4A, Table 1) [20], [21], [22]. In contrast, the β-catenin-negative MDA-MB-453 cell line responds to IC261 with an IC50 of 86 uM, more than 100-fold less sensitive than the MCF7 cell line (Fig. 4, Table 1). We also performed IC261 dose response curves in the BT474, BT549, HS578T and SKBR3 cell lines and again found that the β-catenin positive cells responded at low micromolar doses to IC261 while the β-catenin negative line required 10- to 100-fold higher doses of IC261 to inhibit proliferation (Table 1). We also found that non-tumorigenic human mammary epithelial cells immortalized with hTERT (HMEC-hTERT) respond to IC261 similarly to the β-catenin-negative lines, with an IC50 of 46 uM (Table 1). In agreement with these results, we also observed that a second CK1ε inhibitor, PF-670462 [23], preferentially inhibits proliferation of β-catenin-positive MCF7 cells as compared to β-catenin-negative MDA-MB-453 cells, with IC50 values of 6 uM versus 33 uM, respectively (Fig. 4B). Together, these observations suggest that pharmacological inhibition of CK1ε preferentially affects the proliferation of breast cancer cells with aberrant β-catenin activity, compared to both immortalized mammary epithelial cells and breast cancer cells without β-catenin activity.

**Table 1:** IC50 values for growth inhibitory effect of IC261 in breast cancer cell lines.

Cell line	IC261 IC50	β-catenin
MCF7	0.5 uM	+
MDA-MB-453	86 uM	-
BT474	6.9 uM	+
BT549	0.9 uM	+
HS578T	0.2 uM	+
SKBR3	43 uM	-
HMEC-hTERT	46 uM	NA

IC50 values for each cell line were calculated from a representative experiment performed in triplicate using Graphpad Prism. β-catenin status is indicated in right column.

### Loss of *CSNK1E* Function Abrogates β-Catenin Activity

**Figure 5:**
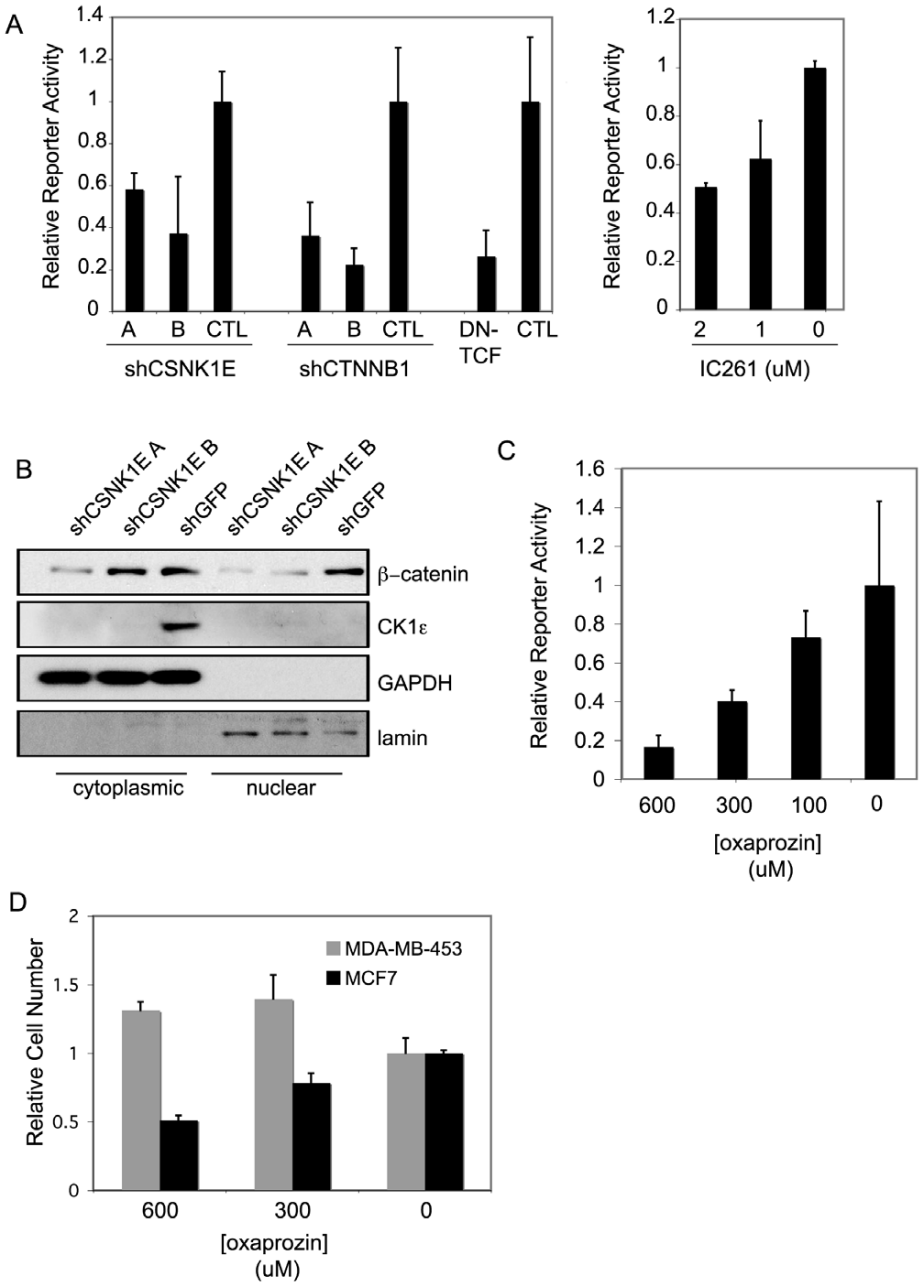
Loss of *CSNK1E* function abrogates Wnt/β-catenin signaling. (A) Effect of RNAi suppression of *CSNK1E* on β-catenin transcriptional reporter activity. 293T cells were cotransfected with a β-catenin expression construct, pRL-SV40, the indicated shRNA construct and the β-catenin-responsive pTOPFLASH reporter constructs. Parallel transfections with pFOPFLASH reporter constructs were performed to normalize for β-catenin-specific activity. *CTNNB1* shRNAs and dominant-negative TCF (DN-TCF) were included for comparison. Graph shows mean ± SD of a representative experiment performed in triplicate. Right panel shows effect of IC261 on β-catenin transcriptional reporter activity. MCF7 cells transfected with pRL-SV40, and either the β-catenin-responsive pTOPFLASH reporter or the control pFOPFLASH reporter, were treated with the indicated concentrations of IC261 for 48 hours. Graph shows mean ± SD of a representative experiment performed in triplicate. (B) Immunoblot analysis of cytoplasmic (left) and nuclear (right) β-catenin levels in MCF7 cells 72 hrs post transduction with shRNA against *CSNK1E*. Immunoblots for cytoplasmic GAPDH and nuclear lamin were performed to verify fractionation. (C) Effect of oxaprozin on β-catenin transcriptional activity after 48 hrs. (D) Differential effects of oxaprozin on relative cell number of β-catenin active cells (MCF7) versus β-catenin inactive cells (MDA-MB-453).

To validate the observation from the β-catenin reporter screen, that *CSNK1E* is required for β-catenin activity, we used the same two *CSNK1E*-specific shRNA sequences that we tested above in an independent β-catenin reporter assay and observed that suppression of *CSNK1E* reduces β-catenin transcriptional activity by more than two-fold (Fig. 5A). This reduction was similar to the effects seen with either β-catenin suppression or expression of a dominant-negative TCF (Fig. 5A). Furthermore, we found that treatment with the CK1ε inhibitor IC261 at 1 or 2 uM reduces β-catenin transcriptional activity by approximately two-fold in MCF7 cells, supporting the hypothesis that IC261 reduces viability at least in part through inhibition of Wnt/β-catenin signaling (Fig. 5A).

To gain insight into the mechanism by which CK1ε regulates β-catenin-driven transcription, we determined the effects of *CSNK1E* suppression on β-catenin levels. We observed a reduction in the levels of nuclear and cytoplasmic β-catenin upon loss of *CSNK1E* expression, suggesting that CK1ε regulates β-catenin stability (Fig. 5B).

To further investigate *CSNK1E* function in an unbiased fashion, we performed microarray-based transcript profiling on MCF7 cells in which *CSNK1E* expression had been suppressed with RNAi. Among the genes downregulated upon *CSNK1E* suppression were many canonical β-catenin target genes, including *MYC*, *CD44*, *BIRC5*, *PPARD*, and *TCF1* (data not shown). From these expression profiles, we used Genepattern [24] to identify comparative markers that distinguish cells transduced with *CSNK1E*-specific shRNA from cells transduced with GFP-specific shRNA to generate a molecular signature of *CSNK1E* suppression. We then used this signature to query the Connectivity MAP (CMAP) database of expression signatures generated from cells treated with chemical compounds [25] and identified two compounds that induced similar expression profile signatures, with multiple instances scoring highly in the comparative analysis, the nonsteroidal anti-inflammatory drug (NSAID) oxaprozin and resveratrol (Fig. S1).

We tested the effects of oxaprozin on β-catenin reporter activity and observed a dose-dependent decrease in activity beginning with 300 uM, the concentration isolated as a match to the signature of MCF7 cells treated with *CSNK1E*-specific RNAi (Fig. 5C). Similar to our observations with *CSNK1E* suppression by RNAi, oxaprozin inhibited the proliferation of the β-catenin-positive cells line, MCF7 (Fig. 5D). The target of oxaprozin in this instance is likely to be COX-1, as MCF7 cells express little or no COX-2 [26] and COX-2 selective inhibitors failed to score as a match to *CSNK1E* suppression in the CMAP analysis. In contrast to MCF7 cells, the MDA-MB-453 cells, which show low levels of β-catenin, are resistant to oxaprozin, consistent with the idea that loss of β-catenin activity underlies the response to oxaprozin (Fig. 5D).

The other compound identified in the CMAP analysis, resveratrol, was recently shown to activate SIRT1, which promotes the deacetylation of β-catenin, ultimately leading to inhibition of transcription [27]. However, in this case, we observe that resveratrol affects the proliferation of both β-catenin positive and negative cells similarly, likely reflecting its ability to impact many cellular processes (Fig. S1B). Notwithstanding the pleiotropic effects of resveratrol on proliferation, our ability to match *CSNK1E* suppression with two compounds that subsequently proved to be inhibitors of β-catenin activity further supports the hypothesis that *CSNK1E* functions in breast cancer cells to promote β-catenin signaling.

### *CSNK1E* Promotes Oncogenic Transformation

**Figure 6:**
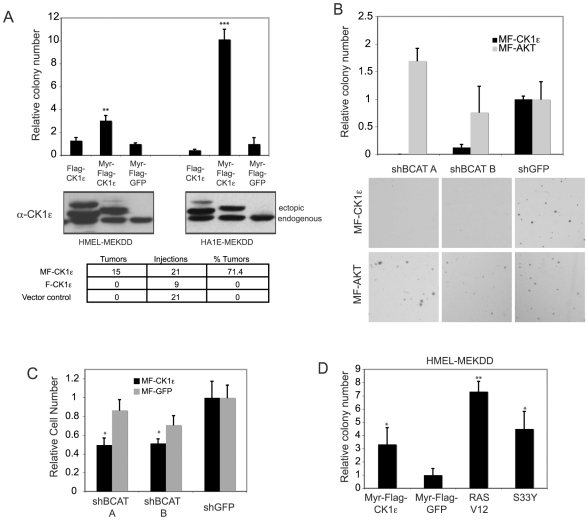
CK1ε expression promotes oncogenic transformation. Anchorage-independent growth of (A) human mammary epithelial or human kidney epithelial cells expressing hTERT, SV40 Early Region, and activated MEK (HMEL-MEKDD or HA1E-MEKDD) with the indicated expression constructs. Colony numbers were normalized to the control and graphs show mean ± SD of a representative experiment performed in triplicate. Immunoblotting with a CK1ε antibody to confirm expression is shown in middle panel. Lower panel shows tumor formation of HA1E-MEKDD cells expressing myristoylated CK1ε. (B) β-catenin is required for anchorage-independent growth of HA1E-MEKDD cells expressing myristoylated CK1ε. Colony numbers were normalized to the GFP control and graph shows mean ± SD of a representative experiment performed in triplicate. Representative images from each condition are shown in lower panel. (C) Differential effect of *CTNNB1* suppression on the proliferation of HA1E-MEKDD cells expressing CK1ε versus GFP control. P values calculated for shBCAT A and shBCAT B for MF-CK1e-expressing cells are 0.034 and 0.038 respectively. P values for shBCAT A and shBCAT B for MF-GFP-expressing cells are 0.33 and 0.10 respectively. (D) Expression of constitutive active S33Y β-catenin mutant can substitute for myristyolated-CK1ε in promoting anchorage-independent growth in human mammary epithelial cells expressing hTERT, SV40 Early Region and activated MEK.

Aberrant activation of β-catenin through dysregulation of components in the Wnt signaling pathway is known to promote tumorigenesis. Having determined that *CSNK1E* regulates Wnt/β-catenin signaling, as well as proliferation of β-catenin-positive breast cancer cells, we next determined whether *CSNK1E* is potentially involved in promoting β-catenin-dependent oncogenic transformation in breast cancer. In prior work, we established human embryonic kidney epithelial cells engineered to express hTERT, the SV40 Early Region and an activated allele of *MEK* as a model system for demonstrating transformation activity of oncogenes, including *AKT1* and *IKBKE* [28]. Using this experimental system, we had observed that expression of myristoylated CK1ε transforms these cells, as assessed by anchorage-independent growth *in vitro*. Here, we confirmed that expression of myristoylated CK1ε induces cell transformation in multiple cell types including human embryonic kidney and human mammary epithelial cells expressing hTERT, the SV40 Early Region, and constitutively active MEK (Fig. 6A), as well as in NIH 3T3 mouse fibroblasts (data not shown). Expression of myristoylated CK1ε is also able to support tumor formation *in vivo*, as assessed by growth of xenografts in an immunodeficient mouse model (Fig. 6A). Interestingly, expression of non-myristoylated CK1ε was unable to promote either anchorage-independent growth or tumor formation *in vivo* (Fig. 6A), consistent with the possibility that membrane localization is required to induce activation of CK1ε, in a manner similar to other ectopically expressed signaling molecules, such as AKT, RAF or PI3-kinase [29], [30], [31] (Fig. 6A).

To determine whether β-catenin activity is essential for the ability of *CSNK1E* to transform human cells, we characterized the effect of loss of β-catenin function in cells transformed by expression of myristoylated CK1ε. We observed that suppression of *CTNNB1* strongly inhibited the ability of *CSNK1E* to promote anchorage-independent growth *in vitro* (Fig. 6B). In contrast, the anchorage-independent growth of cells transformed by AKT is not significantly affected by suppression of β-catenin (Fig. 6B). Moreover, cells transformed by *CSNK1E* demonstrated an enhanced sensitivity to β-catenin suppression compared to control cells, suggesting that the *CSNK1E*-transformed cells develop a dependence on β-catenin function for proliferation (Fig. 6C). To determine whether β-catenin activity may be sufficient to mediate the ability of *CSNK1E* to promote transformation, we replaced *CSNK1E* in the mammary epithelial cell transformation assay with the β-catenin S33Y mutant [32] and found that constitutive activation of β-catenin functionally substitutes for *CSNK1E* in this context (Fig. 6D). Taken together, these data suggest that *CSNK1E* is not only required for cancer cell proliferation and transformation in the context of activated β-catenin, but can also promote transformation, potentially through activation of the Wnt/β-catenin signaling pathway.

## Discussion

Utilizing complementary unbiased functional genomics approaches, we have identified *CSNK1E* as a gene essential for Wnt/β-catenin signaling and survival in a subset of breast cancers that exhibit aberrant β-catenin activity. The role of *CSNK1E* in the regulation of β-catenin activity was corroborated by the identification of compounds that inhibit β-catenin signaling as capable of inducing expression profiles similar to *CSNK1E* suppression by RNAi. Moreover, expression of an activated form of CK1ε converts immortalized human cells to tumorigenicity, in a β-catenin-dependent manner.

The majority of breast tumors exhibit evidence of activation of canonical Wnt/β-catenin signaling and alterations predicted to upregulate Wnt/β-catenin signaling have been reported, including epigenetic silencing of Wnt antagonists *SFRP1* and *WIF1* and upregulation of Wnt ligands and Fzd receptors [7], [8], [33]. These events would promote the establishment of an autocrine Wnt signaling loop that may lead to dependence on continued signaling for viability, resulting in sensitization of these cells to loss of *CSNK1E* function. Our observations suggest that in this context, *CSNK1E* behaves as a synthetic lethal gene to aberrant activation of β-catenin.

Prior reports indicate that CK1ε can phosphorylate and thereby regulate multiple targets in the Wnt signaling cascade, including Dvl, LRP6, APC, Axin and β-catenin [17]. We observed that membrane targeting of CK1ε is required for it to support anchorage-independent growth and that the *APC*-mutated line DU4475 is relatively resistant to *CSNK1E* suppression, implicating a role for *CSNK1E* upstream of *APC*. Here, our findings support a membrane-proximal function for CK1ε, upstream of the degradation complex, consistent with the hypothesis that the relevant target may be membrane-associated, such as Dvl and/or LRP6.

Of potential therapeutic importance, we have demonstrated that cancer cells with aberrant β-catenin activity exhibit greatly increased sensitivity to CK1ε inhibition, mediated either by RNAi or by use of small molecule inhibitors of CK1ε (Figs. 2, 3). The finding that both suppression of CK1ε expression and inhibition of CK1ε kinase activity led to apoptosis in cell lines dependent on β-catenin make it highly likely that the observed phenotype is due to specific effects on CK1ε rather than an off-target effect. Moreover, the ability to potentially inhibit CK1ε using available inhibitors would facilitate downregulation of Wnt/β-catenin signaling in human tumors, which has proven challenging to target pharmacologically. Interestingly, previous reports have also suggested that pharmacological inhibition of CK1ε can induce apoptosis in MEFs, which is enhanced by loss of p53, suggesting a potential role for p53 in regulating apoptosis upon CK1ε suppression [21].

*CSNK1E* was also identified as an essential gene in cancer cell lines from multiple tumor types in addition to breast, including colon, prostate, and brain (data not shown), as well as other cancer cell lines in previously published reports [34], [35]. Of particular interest, Yang et al provide evidence to suggest that *CSNK1E* is a cancer-specific essential gene and further postulate that this may be due to its role in regulation of Per1/2 degradation and circadian cycling [35]. In contrast, we have not observed changes in Per1 levels in breast cancer cells upon *CSNK1E* suppression, and we failed to detect effects on cell viability after Per1 overexpression (data not shown). Given the many functions attributed to CK1ε, it seems likely that CK1ε is an important regulator of several pathways and its function may be dependent on cell context. However, despite the potentially varying mechanisms in different tumor types, these observations further support the view that inhibition of CK1ε may be broadly applicable as a therapeutic strategy for cancer.

In addition to the identification of *CSNK1E* as a novel cancer-relevant gene, these observations demonstrate the power of integrating complementary unbiased functional genomic approaches to identify genes involved in specific cancer pathways. As high-throughput screening technology becomes increasingly available, systematic integration of the resulting phenotypic annotation datasets will likely prove to be of great use in rapidly pinpointing genes that may provide new avenues for targeting essential cancer pathways.

## Materials and Methods

### Cell Culture and Reagents

Cells were cultured in DMEM (MCF7, MDA-MB-231, MDA-MB-453, T47D, HS578T and 293T), RPMI (BT474, BT549 and DU4475) or McCoy's (SKBR3) medium with 10% inactivated fetal bovine serum. All established breast cancer cell lines are commercially available from ATCC. Human mammary epithelial cells were cultured in DMEM/F12 with 10 ng/ml EGF, 10 ug/ml insulin and 0.5 ug/ml hydrocortisone [28]. Human epithelial kidney cells were cultured in MEM with 10% inactivated fetal bovine serum [28]. NIH 3T3 cells were cultured in DMEM with 10% calf serum. Antibodies were purchased for active β-catenin (Millipore), β-catenin, GAPDH, and lamin (Cell Signaling Technologies), CK1ε (BD Transduction Laboratories), Ras and tubulin (Santa Cruz Biotechnology). IC261 was purchased from Merck; oxaprozin and PF-670462 were purchased from Sigma; and resveratrol was a generous gift from David Sinclair.

### Lentiviral RNAi Screens

Cells plated in 384-well microtiter plates were infected with lentiviral shRNA constructs generated by the RNAi Consortium (TRC) as described [36]. Screens were performed in duplicate both in the presence and absence of puromycin to assess infection efficiency. Assays were performed 6 days post infection using Cell Titer Glo (Promega) and raw luminescence scores were converted to B-scores [13]. Software to implement the B-score and various quality and consistency checks were implemented using the R statistical package (www.R-project.org) and BioConductor [37]. The β-catenin transcriptional reporter screen was performed as described [14].

### Cellular Fractionation and Immunoblotting

Cells were fractionated using the Qproteome Cell Compartment kit from Qiagen, according to manufacturer's instructions. For knockdown validation of shRNA constructs by immunoblotting, cells were infected in 6 well plates and lysates were harvested in RIPA buffer containing protease and phosphatase inhibitors three days post-infection.

### Cell Proliferation and Oncogenic Transformation Assays

Cells were plated in 96 well microtiter plates and 24 hours later, infected with shRNA lentiviruses in triplicate, followed by Cell Titer Glo assay (Promega) six days post infection. The TRC IDs corresponding to the shRNA sequences used for theses experiments include: TRCN0000009965 and TRCN0000001834 (against *CSNK1E*), TRCN0000003843 and TRCN0000003845 (against *CTNNB1*), TRCP0000008679 (against *GFP*). For drug response experiments, cells were plated and 24 hours later, treated with the indicated drug concentrations in triplicate for 72 hours. Drug dose response curves were fitted and IC50 values calculated using a non-linear regression model in Graphpad Prism. Retroviral infections, anchorage-independence assays and xenograft assays were performed as described [28]. The pBP-HA-β-catenin S33Y construct has been described previously [38].

### β-Catenin Reporter Assays

Cells were transfected with either the β-catenin-responsive pTOPFLASH or the control pFOPFLASH firefly luciferase reporter construct [14], along with pRL-SV40 expressing renilla luciferase used as transfection control (Promega) and other shRNA or expression constructs as indicated. Where indicated, drug was added 24 hours post transfection. 48 or 72 hours later, plates were assayed using the Dual-Glo kit (Promega).

### Microarray Analysis

RNA was harvested from MCF7 cells infected 48 hours prior with lentiviruses harboring shRNAs against either *CSNK1E* or *GFP* and submitted to the Dana-Farber Cancer Institute Microarray Core Facility for mRNA expression profiling using Affymetrix U133A2.0. Each infection was performed in triplicate. Raw data were processed and comparative markers distinguishing the two classes were identified using Genepattern software [24]. The top 100 overexpressed genes in and the top 100 underexpressed genes *CSNK1E*-suppressed cells were selected to generate a *CSNK1E* loss-of-function expression signature, which was then used to query the CMAP database (http://www.broad.mit.edu/cmap/).

## Supporting Information

Figure S1 (A) Connectivity Map analysis identifies oxaprozin and resveratrol as compounds that cause transcriptional responses similar to CSNK1E suppression in MCF7 cells, with multiple instances scoring highly. The black lines indicate the ranking of the instances for oxaprozin and resveratrol, with the green area indicating samples with positive connectivity and the red area indicating samples with negative connectivity. (B) Effects of resveratrol on relative cell number of MCF7 versus MDA-MB-453 cells.
(0.39 MB TIF)

Table S1 Primary screen B scores for MCF7, MDA-MB-231, MDA-MB-453 AND T47D cell lines. B-scores for all shRNAs tested in the cell viability/proliferation for each of the cell lines screened. All shRNA constructs are listed by their TRC identifier under “clone ID”. Asterisks denote wells that were removed for technical reasons.
(0.74 MB XLS)
