# Supplementary figures and images for: CK1ε Is Required for Breast Cancers Dependent on β-Catenin Activity
Source: PLoS One. 2010 Feb 1;5(2):e8979. doi: 10.1371/journal.pone.0008979 (PMC2813871; doi:10.1371/journal.pone.0008979)

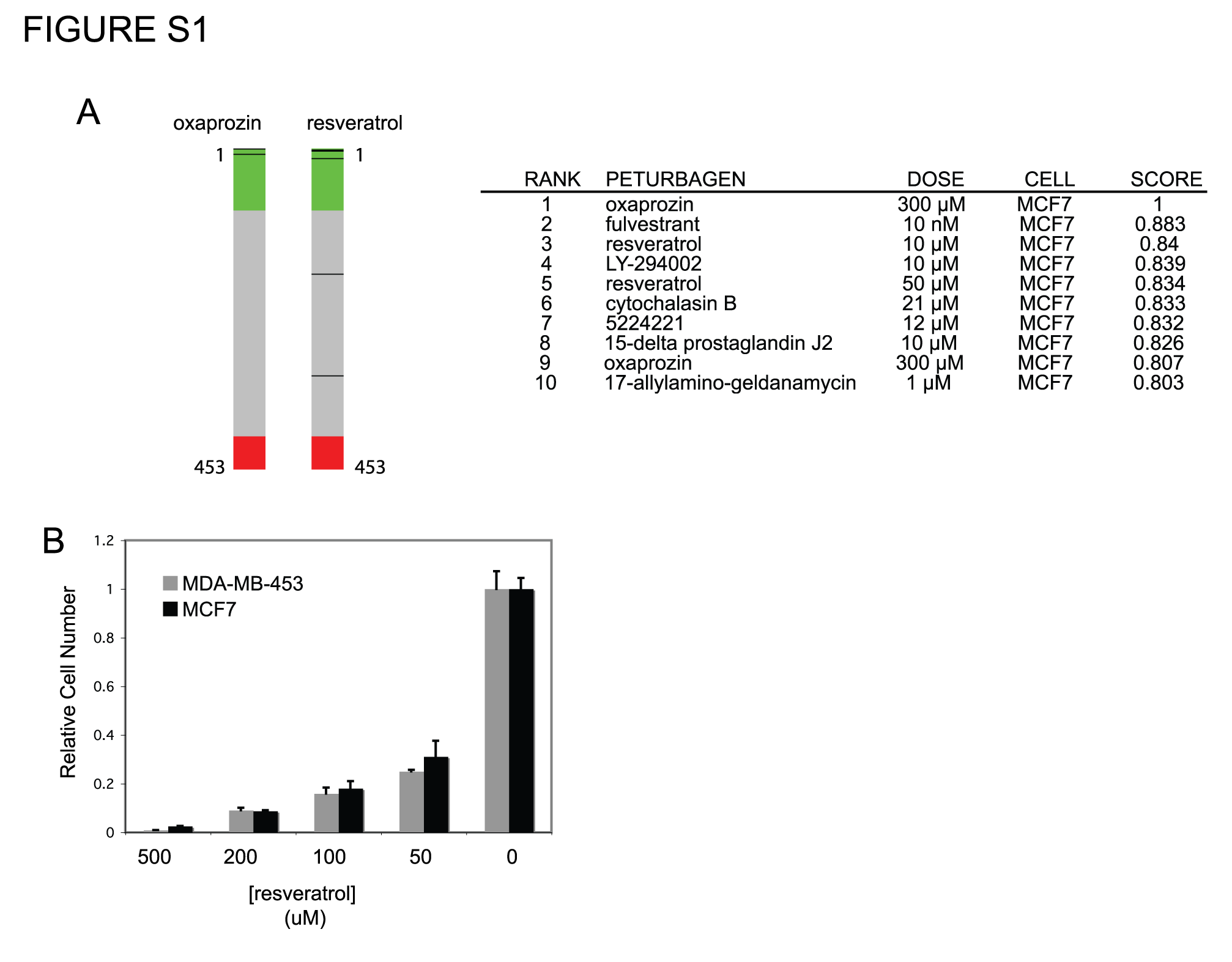

Supplement: Figure S1 — (A) Connectivity Map analysis identifies oxaprozin and resveratrol as compounds that cause transcriptional responses similar to CSNK1E suppression in MCF7 cells, with multiple instances scoring highly. The black lines indicate the ranking of the instances for oxaprozin and resveratrol, with the green area indicating samples with positive connectivity and the red area indicating samples with negative connectivity. (B) Effects of resveratrol on relative cell number of MCF7 versus MDA-MB-453 cells. (0.39 MB TIF) [file pone.0008979.s001.tif]
